# Fecal Elastase Levels Predict Honeycombing in Pancreas Detected with Endoscopic Ultrasound

**DOI:** 10.1155/2018/4625247

**Published:** 2018-12-02

**Authors:** Sinan Akay, Burcu Şirin, Belkıs Ünsal

**Affiliations:** ^1^Katip Celebi University Ataturk Teaching and Research Hospital Gastroenterology Clinic, Izmir, Turkey; ^2^Katip Celebi University Ataturk Teaching and Research Hospital Biochemistry Department, Izmir, Turkey

## Abstract

**Background and Study Aims:**

We aimed to demonstrate the association between fecal elastase levels and Rosemont categories in patients with chronic changes in pancreas detected with endoscopic ultrasound.

**Patients and Methods:**

Patients were selected consecutively from endosonography examinations performed for upper gastrointestinal subepithelial lesions and for pancreas evaluation. Pancreas imaging findings were categorized according to the Rosemont criteria using echoendoscope. Patients who were indeterminate of, suggestive of, and consistent with chronic pancreatitis were included in the study. Fecal elastase measurements were performed after the patients were qualified to participate in the study according to endosonography findings.

**Results:**

Seventy patients were included in the study. 54 of them were male. Mean age of the patients was 51.7 ± 10.2 year. There were 36 patients in the indetermine group for chronic pancreatitis. Mean fecal elastase level was 507.1 ± 14.6 *μ*g/g in the indeterminate group. There were 24 patients in the suggestive group of chronic pancreatitis. Mean fecal elastase level was 400.4 ± 121.4 *μ*g/g in the suggestive group of chronic pancreatitis. There were 10 patients, in the consistent group with chronic pancreatitis. Mean fecal elastase level was 134.8 ± 86.1. The difference between the three groups of fecal elastase values was statistically significant compared with Kruskal Wallis test. Ordinal logistic regression analysis showed that there was a significant relation between endosonografic categories and fecal elastase values with Nagelkerke value of 0.704.

**Conclusions:**

Fecal elastase levels of each of the endosonographic categories were significantly different from each other. Also, fecal elastase values can predict chronic changes in pancreas detected with endoscopic ultrasound.

## 1. Introduction

For over two decades endoscopic ultrasound (EUS) has been used for the investigation of pancreatic diseases and chronic pancreatitis (CP). Its principal use is its competence to detect minor parenchymal and ductal abnormalities that cannot be detected with cross sectional imaging. An assembly of experienced endosonographers in Rosemont, Illinois, in 2007 assessed the literature and form revised diagnostic criteria for EUS for chronic pancreatitis. They assessed three points, pancreas parenchyme, pancreas duct, and correlation with histology, and by combining these they formed an EUS-based diagnostic model. The Rosemont classification is composed of well-defined major and minor criteria and four diagnostic groups. The diagnostic groups include those were were consistent with, suggestive of, and indeterminate for chronic pancreatitis and normal imaging [1, 2].

That consistent with chronic pancreatitis can be formed with 2 major A or 1 major A + 1 major B or 1 major A + >3 minor criteria. That suggestive of chronic pancreatitis can be formed with 1 major A + <3 minor or major B + >3 minor or >5 minor criteria. That indeterminate can be formed with major B alone + <3 minor criteria or 3 to 4 minor features. [1]

Major A criteria include hyperechoic foci (with shadowing) and Major duct calculi (echogenic structure). Major B criterion is lobularity (≥3 contiguous lobules = “honeycombing”). Minor ductal criteria include (1) cyst; (2) dilated duct; (3) irregular duct contour; (4) dilated side branch; (5) hyperechoic duct wall.

Minor parenchymal criteria include (1) hyperechoic strands; (2) hyperechoic foci; (3) lobularity [1].

A tubeless pancreatic function test that measures the content of elastase-1 in stool is used to diagnose functional impairment of the pancreas. FE-1 levels have been shown to have correlation with more correct tests of pancreatic exocrine function, such as the secretin test [3]. Fecal elastase-1 levels have also been shown to have correlation with radiologic tests for chronic pancreatic changes, such as endoscopic retrograde pancreatography [4] and magnetic resonance cholangiopancreatography [5, 6].

In this study, we aimed to demonstrate the relationship between fecal elastase levels and Rosemont categories in patients with chronic changes in the pancreas detected by EUS.

## 2. Patients and Methods

Seventy patients (mean age of 51.7 ± 10.2 years) with chronic changes in the pancreas were diagnosed by endoscopic ultrasound participated in this study. Fifty-four (77%) of the patients were men. Patients were selected consecutively from endosonography examinations performed for upper gastrointestinal subepithelial lesions and pancreas evaluation. Chronic changes in the pancreas were evaluated according to the Rosemont criteria. Patients with consistent, suggestive, and indeterminate chronic pancreatitis were included in the study. Patients with normal pancreas imaging were not included. Cases were included if the endoscopist reported at least three minor criteria for the pancreas.

The study was conducted prospectively between May 2015 and June 2016. This study was approved by the local Ethics Committee. The patients were invited to participate, and those who participated provided written informed consent. Fecal elastase measurements were performed after the patients were qualified to participate in the study according to endosonography findings. Three patients were receiving “pancreas enzyme replacement therapy” that were in the consistent of chronic pancreatitis group.

One experienced endosonographist performed EUS. With patients under conscious sedation, the lineer EG-3870 UTK echoendoscope (Pentax) was used to scan the pancreatic head, body, and tail at 7.5 MHz. The pancreas was examined for the presence/absence of 5 parenchymal (hyperechoic foci with or without shadowing, echogenic stranding, lobularity, honeycombing, and cysts) and 4 ductal (dilation, irregularity, hyperechoic margins, and visible side branches) features.

Stool samples for fecal elastase 1 determination, with firm consistency (not loose or watery), were collected at home or in the hospital. We received the samples the same day and immediately stored them at -20°C. The samples were analyzed for the fecal elastase 1 concentrations by an enzyme linked immunosorbent assay (Bioserv Diagnostics Gmbh) (polyclonal assey). The results were reported in *μ*g/g of stool.

### 2.1. Statistical Analysis

Descriptive statistics were computed for all variables, including means, standard deviations for continuous variables, frequencies, and percentages for categorical factors. The nonparametric Kruskal-Wallis tests were used for continuous factors. Ordinal regression analysis was performed to demonstrate the correlation between endosonography findings and fecal elastase levels. A p value < 0.05 was considered statistically significant. Statistical analyses were performed using Statistical Package for Social Sciences version 20.0 software (IBM Corp.; Armonk, NY, USA)

## 3. Results

Seventy patients were included in the study, 54 of which were male. The mean age of the patients was 51.7 ± 10.2 years. None of the patients was reported alcohol dependence or alcohol consumption beyond social alcohol consumption.

**Figure 1:**
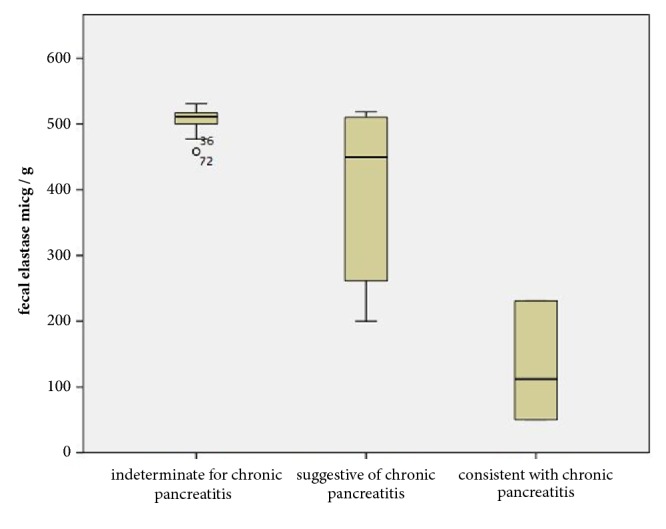
Boxplot of fecal elastase levels grouped according to the EUS Rosemont criteria.

There were 36 patients in the indeterminate for chronic pancreatitis group, and the mean fecal elastase level was 507.1 ± 14.6 *μ*g/g. There were 24 patients in the suggestive of chronic pancreatitis group, and the mean fecal elastase level was 400.4 ± 121.4 *μ*g/g. There were 10 patients in the consistent with chronic pancreatitis group, and the mean fecal elastase level was 134.8 ± 86.1 *μ*g/g. The difference in the fecal elastase values between the three groups was statistically significant when compared with the Kruskal Wallis test. Differences between the groups after Bonferroni correction with the Mann-Whitney U test were also statistically significant (Table 1, Figure 1).

Ordinal logistic regression analysis showed that there was a significant relation between endosonographic categories and fecal elastase values with a Nagelkerke value of 0.704.

**Figure 2:**
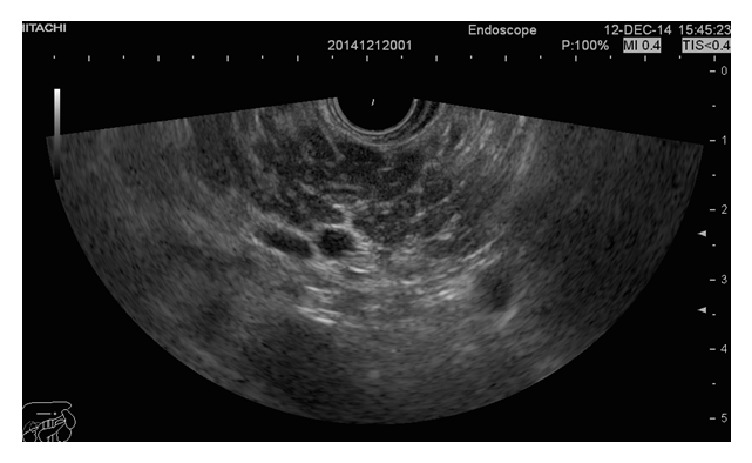
Honeycombing and hypoechoic appearance of pancreas with linear EUS.

**Figure 3:**
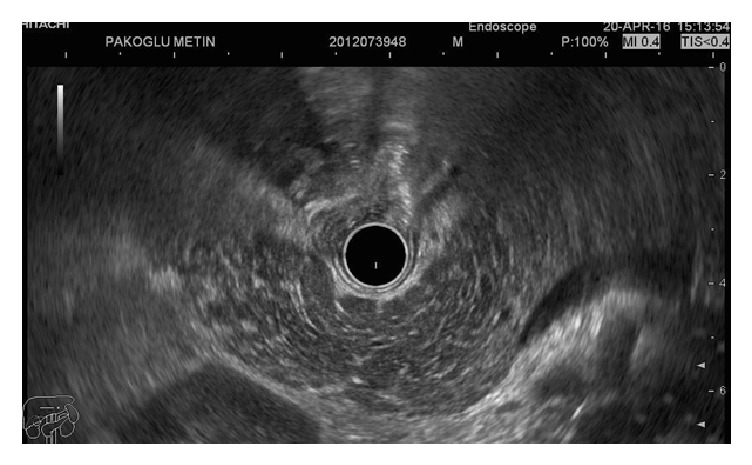
A radial endosonographic image for indeterminate for chronic pancreatitis.

All of the patients in the suggestive of chronic pancreatitis group had a honeycombing appearance with more than 3 minor features (Figure 2). An endosonographic image for indeterminate for chronic pancreatitis is depicted in Figure 3. Patients who were classified in suggestive of chronic pancreatitis group and had honeycombing had more hypoechoic parenchymal appearance.

## 4. Discussion

This study investigates fecal elastase levels in a group of patients with a variety of pancreatic changes using EUS.

A major problem among endosonographers is that “counting criteria” accept that the criteria (e.g., parenchymal cysts versus strands) have equal weight, which is probably not realistic, as the relative weight of each criterion is likely not the same. However one classification system, the Rosemont criteria, attributes relative weights of importance depending on the type of feature (i.e., lobularity is considered a major feature of CP). This is why we used the Rosemont criteria in this study.

In the Rosemont criteria definitions, 3 different criteria are included in the suggestive of chronic pancreatitis group: (1) 1 major A feature + <3 minor features; (2) 1 major B feature + >3 minor features; (3) >5 minor features. [1] In our study, all of the patients in the suggestive group of chronic pancreatitis group showed 1 major B + >3 minor features (honeycombing). The honeycombing appearance, as shown in Figure 2, has a rather hypoechoic and larger parenchymal appearance, implying inflammation in the pancreas parenchyme. This may be the reason for the lower fecal elastase levels in this group.

Fecal elastase levels lower than 200 *μ*g/g are suggestive of exocrine pancreatic insufficiency. However, patients with higher than 200 *μ*g/g fecal elastase levels may not have chronic pancreatitis, although it is not guaranteed that these patients have normal pancreatic function [7, 8]. Findings in our study indicate that patients with a honeycomb pancreas appearance on EUS (suggestive of chronic pancreatitis) have significantly lower fecal elastase levels than patients in the indeterminate group. The clinical significance of the honeycomb pancreas is not delineated clearly in the literature, but in our study, all of the patients in this group had a large and hypoechoic pancreas, indicating inflammation. We may further speculate that these patients have deteriorating pancreatic function and have inflammation in the parenchyme.

This study does have limitations. The study was performed at a single academic medical center whose practice may not be generalizable to EUS practice at other centers. The small sample size limited subgroup analyses. For the Rosemont criteria, the study also involved a relatively complex algorithm to stratify the multiple categories. However, the endosonographer became accustomed to the procedures and after a while the patients are categorized spontaneously into one group. Although, for chronic pancreatitis Rosemont classification is the most recent and detailed one, it is not validated yet. However, in order to validate this, comparison to gold standard pancreas biopsy is technically not easy because of the risks and difficulty in obtaining pancreas biopsy. Also, in chronic pancreatitis obtained biopsy may not represent whole pancreas because of sampling error [9, 10].

There are validated commercial fecal elastase ELISA kits using monoclonal (ScheBo Biotech) or polyclonal antibodies (Bioserv Diagnostic) [11]. Several clinical studies have compared the diagnostic efficiency of monoclonal and polyclonal antibodies based assays [12]. Polyclonal antibody test using 2 different polyclonal antisera to human elastase has been reported to be positive in 78% of patients compared with 69% positivity for the monoclonal test in the same patients at a cut-off 200 *μ*g/g elastase [13]. Also, binding studies showed that polyclonal test seems to detect antigens that partly differ from classic elastase [14]. Simultaneous evaluation of stool specimens showed a tendency for higher values in polyclonal test, which might cause a higher proportion of false negative results. The weakness of the polyclonal test for measuring fecal elastase is that patients have to discontinue exogenous PERT for testing. In our study, we used polyclonal test, and only 3 of the patients were receiving PERT who had consistent with chronic pancreatitis in EUS, and their fecal elastase 1 levels were measured lower than 200 *μ*g/g which had no effect on statistical findings.

Recently, endoscopic pancreatic function test (ePFT) using secretin stimulation has made direct pancreatic function testing more accessible and time efficient allowing clinicians the opportunity to perform a functional assessment of pancreas with ease. While the ePFT is being used more often in diagnosis, it does have the drawback of being time intensive and availability at only specialized centers [15]. One study found that 30 and 45 minute bicarbonate measurements had the highest agreement with the full test and had a specificity of 93% for diagnosing CP [16]. In another study, endoscopic ultrasound evaluation of pancreatic duct compliance following secretin stimulation along with EUS morphologic examination and duodenal fluid (HCO3) measurement (ePFT) in one endoscopic session was performed and reported positive correlation between pancreatic ductal compliance and duodenal fluid (HCO3) [17].

In conclusion, more large-scale, longitudinal studies are needed to demonstrate the relation between endosonographic findings and fecal elastase levels in patients with chronic changes in the pancreas. We need to follow those patients who were suggestive of chronic pancreatitis to document pancreas deterioration. For fecal elastase levels, a new cut-off level demonstrating normal pancreas exocrine function should be determined.

## Figures and Tables

**Table 1:** Descriptive data according to fecal elastase levels of the study population.

	N	mean fecal elastase +/- sd *μ*g/g	minimum *μ*g/g	maximum *μ*g/g
Indeterminate	36	504 ± 18	458	531
Suggestive	24	400 ± 121	200	519
Consistent	10	134 ± 86	50	213

## Data Availability

The data used to support the findings of this study are available from the corresponding author upon request.
